# The Routine Follow-up Head CT: Is it Still a Necessary Step in the Thrombolysis Pathway?

**DOI:** 10.1007/s12028-021-01348-4

**Published:** 2021-09-27

**Authors:** Edward J. Llinas, Alexandra Max, Sheena Khan, Elisabeth B. Marsh

**Affiliations:** Department of Neurology, School of Medicine, Johns Hopkins University, 600 North Wolfe St. Phipps 446C, Baltimore, MD 21287 USA

**Keywords:** Stroke, Treatment, Optimization, Outcomes, Computed tomography, Protocol

## Abstract

**Background:**

The 24-h head computed tomography (CT) scan following intravenous tissue plasminogen activator or mechanical thrombectomy (MT) is currently part of most acute stroke protocols. However, as evidence emerges regarding who is at highest risk for treatment complications, the utility of routine neuroimaging for all patients has become less clear.

**Methods:**

Four hundred seventy-five patients presenting with acute ischemic stroke to Johns Hopkins Bayview Medical Center between 2004 and 2018 and treated with intravenous tissue plasminogen activator and/or MT were evaluated. Neuroimaging performed during the first 48 h of hospitalization was reviewed for edema, hemorrhagic transformation (HT), or other findings altering management. Early imaging (< 24 h), performed for neurologic deterioration, was compared with imaging performed per protocol (24 ± 6 h). Factors predictive of radiographically and clinically significant findings on per-protocol imaging were determined.

**Results:**

One hundred fifty-three patients (32%) underwent early imaging. These patients generally had more severe strokes. HT was found in 15% of cases. For the remaining patients (*n* = 322), imaging at 24 h impacted acute management for only 24 patients: resulting in emergent hemicraniectomy in 1 (0.3%) and leading to additional imaging to monitor asymptomatic HT or edema in 23 (7.1%). Advanced age, higher stroke severity, MT, and atrial fibrillation were associated with significant findings on the 24-h CT scan. Only 2 of the 24 patients had an initial National Institutes of Health Stroke Scale score of < 7.

**Conclusions:**

The 24-h head CT scan does not change management for most patients, particularly those with low National Institutes of Health Stroke Scale scores who do not undergo MT. Consideration should be given to removing routine follow-up imaging from postthrombolysis protocols in favor of an examination-based approach.

## Introduction

In 1995, tissue plasminogen activator (tPA) was first shown to significantly improve outcomes for patients presenting with acute ischemic stroke [1]. Advancements in technology have resulted in extension of the treatment window and augmentation of recanalization with mechanical thrombectomy (MT) [2]. However, reperfusion of injured tissue confers risk of hemorrhagic transformation (HT) and reperfusion injury [1]. Therefore, patients who receive intravenous (IV) tPA or MT are typically monitored in an intensive-care-like setting (intensive care unit or intermediate care unit) for 24 h post treatment to detect signs of stroke progression, symptomatic HT, and herniation. A routine 24-h noncontrast head computed tomography (CT) scan was part of the original postthrombolysis protocol [1] and is currently recommended in the National Institute of Neurological Disorders and Stroke and American Heart Association guidelines for acute stroke treatment [3] to radiographically monitor for worsening [3] and predict the likelihood of future stroke [4].

As our knowledge surrounding the risks and benefits of thrombolysis improves, treatment protocols for acute ischemic stroke have evolved, not only extending treatment windows [2] but also including elderly patients [5] and no longer requiring preintervention coagulation studies [6]. The risk of HT is relatively small [1], especially in those with low initial National Institutes of Health Stroke Scale (NIHSS) scores, and typically occurs within the first 12 h of admission [7], but we can now further stratify an individual’s risk for treatment complications, recognizing that factors such as stroke size, age, and renal function are important to consider [8]. In this setting, it is possible that, despite the original benefits of a 24-h noncontrast head CT scan, it may no longer be necessary for all patients, as it exposes them to additional radiation [9], increases health care costs, and delays patient throughput. American Heart Association guidelines are being adapted to include consideration of magnetic resonance imaging (MRI) as an alternative imaging modality to CT; however, a majority of institutions continue to use the more traditional approach, and nearly all continue to rely on some form of 24-h neuroimaging to make triage decisions regarding appropriate level of care. This may be unnecessary, and an approach to order follow-up CT imaging based on changes in neurological examination fundings may be more appropriate, with MRI obtained at some point during the hospitalization as part of the standard stroke workup, but not required at 24 h, to determine final size and etiology of the infarct.

This study evaluates the utility of the routine 24-h noncontrast head CT scan as part of the postthrombolysis protocol by identifying the proportion of per-protocol neuroimaging studies resulting in alterations to patient management. In addition, we identify factors associated with clinically significant scans that could help to tailor this practice to the individuals most likely to benefit.

## Methods

A prospectively collected cohort of consecutive patients presenting with acute ischemic stroke to the Johns Hopkins Bayview Medical Center between October 2004 and June 2018 who were treated with IV tPA with or without MT was recruited into our institutional-review-board-approved stroke registry. For this study, approved by our institutional review board, we retrospectively determined the significance of the 24-h head CT scan within the cohort. Given the observational nature, informed consent was not required. Information regarding patient demographics (age, race, sex), past medical history (atrial fibrillation, smoking, diabetes, prescribed use of antiplatelets or anticoagulants), stroke characteristics (admission NIHSS score, stroke volume, degree of white matter disease [10]), treatment details (IV tPA, MT, time to thrombolysis), and medical variables (admission systolic blood pressure, peak systolic blood pressure, serum creatinine level, platelet count, low-density lipoprotein level, international normalized ratio) was obtained through chart review.

### Neuroimaging

Radiology reports of all neuroimaging (CT or MRI) performed during the first 48 h of hospitalization were reviewed for the presence of edema, mass effect, herniation, stroke evolution or progression, and hemorrhage. Hypodensities due to ischemia versus those due to edema were differentiated by the presence of mass effect or herniation. Findings, along with infarct location and white matter grading, were confirmed by a board-certified vascular neurologist. Carestream [11], available on our institution’s picture archive and communication system (PACS), was used to automatically calculate infarct volume by using diffusion-weighted imaging sequences for patients with MRI (*n* = 335). On the basis of time of thrombolysis and subsequent neuroimaging, patients were dichotomized into those undergoing early CT (< 24 h) versus per-protocol (~ 24 h) imaging. Early CT scans were ordered following assessment by the provider, who was notified by nursing for any change in neurological examination findings. Per-protocol scans were performed 24 ± 6 h post treatment as part of the postthrombolysis pathway.

Imaging was categorized into four groups: no acute abnormality/normal CT findings, expected evolution of stroke (hypodensity without significant surrounding edema or mass effect), appearance of edema/mass effect, and presence of hemorrhage. This categorization was confirmed by a board-certified vascular neurologist. Patients with evidence of edema with mass effect or HT were considered to have radiographically significant findings. Additional chart review was performed to determine clinical significance: if imaging changed management, resulting in additional imaging for monitoring, an emergent procedure (such as external ventricular drain or hemicraniectomy), administration of hypertonic saline or mannitol, or alteration in blood pressure goals. Daily progress notes were reviewed for evidence of alterations in goals due to imaging findings. Hemorrhages were further classified as asymptomatic HT or symptomatic intracranial hemorrhage (sICH), defined as the presence of blood on neuroimaging findings corresponding to a clinical deterioration [1].

### Statistics

Data were analyzed by using Stata version 14 (Stata Corp, College Station, TX). Differences between those undergoing early CT versus per-protocol imaging, along with differences in the rates of sICH, were compared using Student’s *t*-tests and χ^2^ analyses for continuous and categorical variables, respectively. Those undergoing early CT imaging were then removed from analysis, and only those completing per-protocol imaging were reviewed for subsequent analyses. The primary outcome of interest was the percentage of patients undergoing per-protocol imaging that was (1) radiographically or (2) clinically significant. Student’s *t*-tests (continuous variables) and *χ*^2^ analyses (categorical variables) were used to determine factors associated with imaging of radiographic or clinical significance. A *p* value equal to or less than 0.05 was considered statistically significant.

## Results

Four hundred seventy-five patients were admitted and met criteria for inclusion over the study period. Three hundred ninety-eight had been treated with IV tPA alone, 36 with MT alone, and 41 with combination therapy. The average age of the cohort was 67.2 years (SD = 16.3). Fifty-five percent (*n* = 260) were women, and 28% (*n* = 133) were African American.

### Early Imaging Differences

One hundred fifty-three patients (32.2%) underwent early imaging following thrombolysis due to a change in clinical examination findings. The average time to early imaging was 10.7 h (SD = 5.7). On average, patients who underwent imaging early had greater stroke severity (mean admission NIHSS score 12.5 [SD = 7.1] versus 9.9 [SD = 7.0], *p* < 0.001; mean infarct volume 81.4 ml [SD = 125.8] versus 37.4 ml [SD = 66.1], *p* < 0.001) than those who underwent imaging per protocol. Patients with an early CT scan were also more likely to have atrial fibrillation (34.0% versus 20.5%, *p* = 0.001) and a history of smoking (63.1% versus 52.5%, *p* = 0.042). Symptomatic hemorrhage rates were significantly higher in the early imaging group (15.0% versus 0.3%, *p* < 0.001), and they required longer hospital stays (9.1 [SD = 8.5] versus 6.5 days [SD = 6.4], *p* < 0.001). See Table 1 for further details.

**Table 1:** Patient characteristics

	Total cohort (*N* = 475)	Early CT (*n* = 153)	Per protocol (*n* = 322)	*p* value
Demographics				
Age, mean (SD) (yr)	67.2 (16.3)	68.6 (15.5)	66.6 (16.7)	0.223
Race (Black), *n* (%)	133 (28.0)	35 (22.9)	98 (30.4)	0.086
Sex (male), *n* (%)	215 (45.3)	67 (43.8)	148 (46.0)	0.657
Medical history				
Atrial fibrillation, %	24.8	34.0	20.5	0.001
Smoking, %	55.7	63.1	52.5	0.042
Diabetes, %	27.8	30.7	26.4	0.326
Antiplatelet, %	38.0	38.1	37.9	0.981
Anticoagulant, %	8.7	9.7	8.7	0.730
Medical variables				
Admission SBP, mean (SD) (mm Hg)	156.5 (28.7)	156.4 (27.7)	156.6 (29.1)	0.960
Peak SBP, mean (SD) (mm Hg)	179.2 (25.7)	182.2 (27.6)	177.8 (24.7)	0.093
Creatinine, mean (SD) (mg/dl)	1.26 (1.1)	1.27 (1.2)	1.25 (1.0)	0.803
Platelet count, mean (SD) (cells per μl)	235.9 (78.3)	240.3 (78.5)	233.8 (78.3)	0.398
LDL, mean (SD) (mg/dl)	97.4 (41.8)	96.8 (39.8)	97.7 (42.8)	0.831
INR, mean (SD)	1.10 (0.4)	1.15 (0.7)	1.07 (0.2)	0.068
Stroke characteristics				
Admission NIHSS score, mean (SD)	10.7 (7.1)	12.5 (7.1)	9.9 (7.0)	< 0.001
Stroke volume, mean (SD), ml	52.4 (93.1)	81.4 (125.8)	37.4 (66.1)	< 0.001
CHS score (white matter grade), mean (SD)	3.34 (2.4)	3.00 (2.4)	3.48 (2.4)	0.085
Treatment				
Time to treat, mean (SD) (h)	2.83 (2.2)	2.88 (2.9)	2.80 (1.8)	0.721
Time to 24-h CT scan, mean (SD) (h)			25.3 (7.7)	
Time to early CT scan, mean (SD) (h)		10.7 (5.7)		
IV tPA only, *n* (%)	398 (83.8)	124 (81.0)	274 (85.1)	0.263
MT only, *n* (%)	36 (7.6)	8 (5.2)	28 (8.7)	0.132
IV tPA and MT combination, *n* (%)	41 (8.6)	22 (14.4)	19 (5.9)	0.002
Outcomes				
Length of stay, mean (SD) (d)	7.29 (7.2)	9.06 (8.5)	6.45 (6.4)	< 0.001
Asymptomatic ICH, *n* (%)	81 (17.1)	35 (22.9)	46 (14.3)	< 0.001
Symptomatic ICH, *n* (%)	24 (5.1)	23 (15.0)	1 (0.3)	< 0.001

*CHS* cardiovascular health study, *CT* computed tomography, *ICH* intracranial hemorrhage, *INR* international normalized ratio, *IV tPA* intravenous tissue plasminogen activator, *LDL* low-density lipoprotein, *NIHSS* National Institutes of Health Stroke Scale, *MT* mechanical thrombectomy, *SBP* systolic blood pressure

### Per-protocol Cohort

#### Clinically Significant Imaging

The remaining patients (*n* = 322) were scanned per protocol an average of 25.3 h (SD = 7.7) post thrombolysis. Of these individuals, only one (0.3%) had CT findings that significantly altered care. This patient had a large stroke (final volume 324 ml) with a consistently poor examination funding (admission NIHSS score 9, right middle cerebral artery syndrome) but was taken for emergent hemicraniectomy when the 24-h head CT scan revealed increased edema and blood products. No other patients required hypertonic solutions or other interventions as a direct result of the routine imaging.

Twenty-three (7.1%) patients required additional follow-up scans to monitor for HT or edema. In general, patients who required additional neuroimaging for follow-up monitoring were older (mean age 73.8 years [SD = 15.3] versus 66.1 years [SD = 16.8], *p* = 0.032) and had a history of atrial fibrillation (43.5% versus 18.9%, *p* = 0.005) and anticoagulant use (27.3% versus 7.3%, *p* = 0.001) (see Table 2 for full details). They presented with more severe strokes (admission NIHSS score 14.7 [SD = 5.9] versus 9.51 [SD = 6.9], *p* = 0.001; mean stroke volume 102.6 ml [SD = 111.8] versus 32.4 ml [SD = 58.9], *p* = 0.000) and were more likely to have been treated with MT (34.8% versus 6.4%, *p* = 0.000) than those not requiring additional monitoring. When patients treated with IV tPA alone who underwent routine imaging (*n* = 274) were considered, only 14 of the per-protocol CT scans led to additional imaging. Only one of these individuals had an NIHSS score of < 7.

**Table 2:** Predictors of significant imaging

	Clinically significant (additional imaging required)			Radiographically significant		
	Yes (*n* = 23)	No (*n* = 299)	*p* value	Yes (*n* = 65)	No (*n* = 257)	*p* value
Demographics						
Age, mean (SD) (year)	73.8 (15.3)	66.1 (16.8)	0.032	71.4 (17.0)	65.4 (16.5)	0.009
Race (Black), *n* (%)	2 (8.7)	95 (32.1)	0.019	8 (12.3)	90 (35.0)	< 0.001
Sex (male), *n* (%)	10 (43.5)	137 (46.3)	0.795	28 (43.1)	120 (46.7)	0.601
Medical history						
Atrial fibrillation, %	43.5	18.9	0.005	27.7	18.7	0.108
Smoking, %	47.6	53.4	0.608	46.6	53.9	0.315
Diabetes, %	21.7	26.4	0.627	27.7	26.1	0.791
Antiplatelet, %	45.5	37.1	0.434	45.2	36.1	0.190
Anticoagulant, %	27.3	7.3	0.001	17.7	6.4	0.005
Medical variables						
Admission SBP, mean (SD) (mm Hg)	153.2 (23.9)	156.6 (29.5)	0.583	157.8 (30.1)	156.3 (28.9)	0.705
Peak SBP, mean (SD) (mm Hg)	182.1 (22.8)	177.3 (24.9)	0.368	179.3 (26.0)	177.4 (24.4)	0.583
Creatinine, mean (SD) (mg/dl)	1.23 (0.4)	1.25 (1.0)	0.934	1.28 (0.7)	1.24 (1.1)	0.784
Platelet count, mean (SD) (cells per μl)	203.7 (72.6)	236.2 (78.7)	0.056	218.6 (72.0)	237.6 (79.5)	0.080
LDL, mean (SD) (mg/dl)	87.2 (50.6)	98.0 (42.1)	0.242	95.9 (41.4)	98.1 (43.2)	0.720
INR, mean (SD)	1.14 (0.28)	1.07 (0.13)	0.027	1.11 (0.2)	1.06 (0.1)	0.018
Stroke characteristics						
NIHSS score, mean (SD)	14.7 (5.9)	9.51 (6.9)	0.001	13.9 (7.0)	8.87 (6.6)	< 0.001
CHS score (white matter grade), mean (SD)	3.87 (2.3)	3.45 (2.4)	0.520	3.33 (2.2)	3.51 (2.4)	0.651
Stroke volume, mean (SD) (ml)	102.6 (111.8)	32.4 (58.9)	< 0.001	87.7 (92.6)	23.1 (47.8)	< 0.001
Treatment						
Time to treat, mean (SD) (h)	2.68 (1.5)	2.81 (1.8)	0.780	2.41 (1.2)	2.88 (1.9)	0.080
Time to 24-h CT scan, mean (SD) (h)	23.8 (2.9)	25.4 (8.0)	0.420	24.9 (5.1)	25.4 (8.2)	0.679
IV tPA only, *n* (%)	14 (60.9)	257 (86.0)	0.001	47 (72.3)	227 (88.3)	0.001
MT only, *n* (%)	8 (34.8)	19 (6.4)	< 0.001	15 (23.1)	12 (4.7)	< 0.001
IV tPA and MT combination, *n* (%)	1 (4.3)	18 (6.0)	0.735	3 (4.6)	16 (6.2)	0.623
Outcomes						
Length of stay, mean (SD) (d)	10.7 (6.1)	6.09 (6.4)	0.001	9.14 (7.2)	5.77 (6.0)	< 0.001
Asymptomatic ICH, *n* (%)	15 (65.2)	31 (10.4)	< 0.001	40 (61.5)	6 (2.3)	< 0.001
Symptomatic ICH, *n* (%)	1 (4.3)	0 (0.0)	< 0.001	1 (1.5)	0 (0.0)	0.046

*CHS* cardiovascular health study, *CT* computed tomography, *ICH* intracranial hemorrhage, *INR* international normalized ratio, *IV tPA* intravenous tissue plasminogen activator, *LDL* low-density lipoprotein, *NIHSS* National Institutes of Health Stroke Scale, *MT* mechanical thrombectomy, *SBP* systolic blood pressure

#### Radiographically Significant Imaging

One hundred fourteen patients (35.4%) had normal head CT findings, without evidence of infarct on follow-up imaging, whereas 145 (45.0%) had expected evolution of their stroke. Twenty-six (8.1%) patients showed increasing edema with some mass effect; 22 (6.8%), some evidence of HT; and 15 (4.7%), a combination of both, although only the patient described above was deemed to have an sICH. The average NIHSS score for those with radiographically significant scan findings was 14. Patients with radiographically significant abnormalities on imaging were similar to those requiring additional imaging (Table 2). Although not all patients with evidence of edema or HT underwent reimaging (*n* = 20 of 65), the majority of patients who underwent reimaging had what we defined as radiographically significant CT scan findings (*n* = 20 of 23).

## Discussion

The 24-h head CT scan was an integral component of the early IV tPA trials to evaluate for HT and monitor for edema. It has been important in helping physician teams to feel comfortable downgrading patients post thrombolysis and gauge infarct evolution. However, over time, our knowledge surrounding the risks and benefits of treatment, along with individuals at highest risk for complications, has improved, resulting in changes to acute stroke protocols, for example, with respect to inclusion criteria and required pretreatment testing [5, 6]. Our study suggests it may also be time to reconsider our posttreatment monitoring practices, as only a small number of patients benefit from a routine 24-h noncontrast head CT scan post thrombolysis. This number decreases further if patients do not have a history of atrial fibrillation, have high admission NIHSS scores, or undergo MT. These findings are consistent with those in a previously published study evaluating 131 patients post IV tPA that found that the findings of a 24-h head CT scan resulted in a change in management (decreased blood pressure goal) for only one patient [12]. A second study evaluating 280 patients from the national Get With the Guidelines Database similarly showed that findings of the 24-h head CT scan had no clinical impact for 95% of asymptomatic patients [13]. Our study is larger and also includes patients post thrombectomy, given that this has now become standard of care for large vessel occlusions [14–17], and relies on similar posttreatment protocols yet arrives at the same conclusion, suggesting generalizability across stroke centers and independent of method of recanalization. A third study of 100 patients undergoing treatment with IV tPA and/or MT also found that the vast majority of patients with symptomatic hemorrhage underwent imaging outside of routine monitoring because of changes in examination findings [18]. Our study goes one step further, evaluating not only the utility of routine head CT in detecting HT but also its role in alteration of clinical management.

Consistent with the results of others [12], within our cohort, the findings of the 24-h head CT scan resulted in a true change in management for only one patient (0.3%), a 55-year-old man who presented to our comprehensive stroke center with a large vessel occlusion and underwent MT. After ~ 24 h of a persistently poor examination finding, follow-up imaging revealed a large infarct, increasing mass effect, midline shift, and blood products leading to emergent hemicraniectomy. As this case illustrates, monitoring for evolution of large strokes in the days following thrombectomy is important; however, even in these cases, the 24-h follow-up CT image itself may typically be less clinically useful if there is no degradation of examination findings, given that worsening edema peaks 48–72 h post infarct [19].

Although edema tends to occur days after recanalization [19], HT typically occurs much earlier, within the first 8–12 h of reperfusion [7]. Associated by definition with a change in examination findings, these patients were almost exclusively part of the early imaging cohort in our study. The rate of early CT imaging due to neurological decline was higher than previously reported (30% versus 20%) and may be the result of including patients post MT, which carries an increased risk of HT [20]. Findings suggest that the 24-h scan may miss the critical time window (falling after the highest risk of HT but before delayed edema).

Although need for a routine head CT scan at 24 h was rare, we were able to identify factors potentially associated with both clinical and radiographic significance, allowing clinicians to tailor protocols to address highest-risk patients. Not surprisingly, stroke volume, high NIHSS scores, and atrial fibrillation, all associated with larger, more severe strokes, were significant. This is consistent with the literature that more severe strokes are at higher risk for both early and delayed complications [21], likely because of the friability of the tissue involved, propensity for reperfusion injury, and potential for edema due to damaged tissue. Anticoagulation is most commonly used to treat atrial fibrillation in this population, likely leading to its significance; although it could theoretically have also increased the risk for HT. Similarly, those undergoing MT were at greater risk, likely given the larger stroke size, but also possibly because of vessel manipulation. Prior studies have shown higher rates of both asymptomatic and symptomatic hemorrhage in this group [14–17, 20].

It is also important to point out that for some patients, the 24-h head CT scan may prove reassuring to their treating physicians that current treatment protocols are adequate or that the patient is ready to be downgraded to a lower level of care. However, we would argue that, given the data, choosing an alternative time to imaging, such as 48 h post infarct, when risk for cerebral edema is greater, or opting to use the clinical examination findings alone, particularly for those with smaller strokes, may be more appropriate.

Although there may be some patients who benefit from a 24-h routine head CT scan post thrombolysis, our study illustrates that for the majority of individuals, it is likely unnecessary, along with other modalities (MRI). The current need for routine neuroimaging often keeps individuals in the intensive care unit, even when clinically appropriate for downgrading. This delay can impact both patient care and hospital throughput. In addition, frequent head CT scans increase radiation exposure, which can increase risk of future cancer [9] diagnosis, and increases health care costs. Although these considerations should certainly not be the only factors dictating care, for patients with low NIHSS scores who are neurologically stable, consideration should be given to forgoing the 24-h CT in favor of MRI performed electively at some point during the hospitalization as part of the stroke workup.

Our study is not without limitations. It included a cohort of patients from a single urban comprehensive stroke center. However, findings are consistent with those of prior studies, suggesting generalizability to other populations. This may not be true for centers that do not routinely obtain MRI as part of the stroke workup; however, we believe this practice to be relatively uncommon and would still advocate for following the clinical examination. In addition, we chose to include patients who had received both IV tPA and MT. Although this resulted in a heterogenous population, these treatments are now routinely performed as the current standard of care and share similar posttreatment monitoring protocols. It is important to point out that there has been a shift in treatment paradigms over time and that rates of MT have increased only recently, biasing our cohort to those treated with IV tPA alone; however, our results were consistent for patients over the entire study period and suggest that the 24-h head CT scan may not be needed for stable patients in either group. We also chose to use radiology reports to screen for radiographic significance and evidence of HT or edema. To avoid concern for inconsistency across radiologists, findings were confirmed by a board-certified neurologist for this study. Finally, because of the retrospective analysis of CT findings, determining whether a CT scan performed around the 24-h posttreatment mark was done for clinical indications or per protocol and whether the routine CT scan directly impacted clinical care could theoretically be difficult. However, extensive chart review was performed to determine the relationship between changes in examination findings, imaging orders, and changes to the treatment plan. In addition, findings for the majority of 24-h CT scans were either entirely normal or showed suspected stroke evolution. The small number of remaining cases was reviewed and discussed among two board-certified neurologists to ensure accuracy and consistency.

Regardless of limitations, our study illustrates that the routine 24-h noncontrast head CT scan ordered per protocol as part of the postthrombolysis protocol at most stroke centers likely provides little additive information for acute management for the majority of patients. A vast majority of patients at risk for adverse events undergo early imaging because of evidence of clinical deterioration. We advocate consideration be given to removing the 24-h scan entirely in favor of an examination-driven approach or, alternatively, tailoring the practice to the groups at highest risk: those of advanced age, with atrial fibrillation, who present with high NIHSS scores, and who undergo MT.
